# Crystal structure of *N* ^1^,*N* ^1^-diethyl-*N* ^4^-[(quinolin-2-yl)methyl­idene]benzene-1,4-di­amine

**DOI:** 10.1107/S2056989014027108

**Published:** 2015-01-01

**Authors:** Md. Serajul Haque Faizi, Nazia Siddiqui, Saleem Javed

**Affiliations:** a Department of Chemistry, Indian Institute of Technology Kanpur, Kanpur, UP 208 016, India; b Department of Chemistry, I.H.S. Khandari, Dr B. R. Ambedkar University, Agra 282 002, India

**Keywords:** crystal structure, benzene-1,4-di­amine, quinoline, C—H⋯π inter­actions, quinolinyl-containing Schiff bases

## Abstract

The title compound, C_20_H_21_N_3_, is non-planar with a dihedral angle between the planes of the quinoline and phenyl­enedi­amine rings of 9.40 (4)°. In the crystal, mol­ecules are connected by C—H⋯π inter­actions, generating a chain extending along the *a*-axis direction. Weak C—H⋯π inter­actions also occur.

## Related literature   

For applications of quinolinyl-containing Schiff bases, see: Das *et al.* (2013 ▸); Jursic *et al.* (2002 ▸); Motswainyana *et al.* (2013 ▸); Song *et al.* (2011 ▸). The present work is part of an ongoing structural study of Schiff base–metal complexes, see: Faizi & Hussain (2014 ▸); Faizi & Sen (2014 ▸); Faizi *et al.* (2014 ▸). For related Schiff bases and their applications, see: Gonzalez *et al.* (2012 ▸); Patra & Goldberg (2003 ▸).
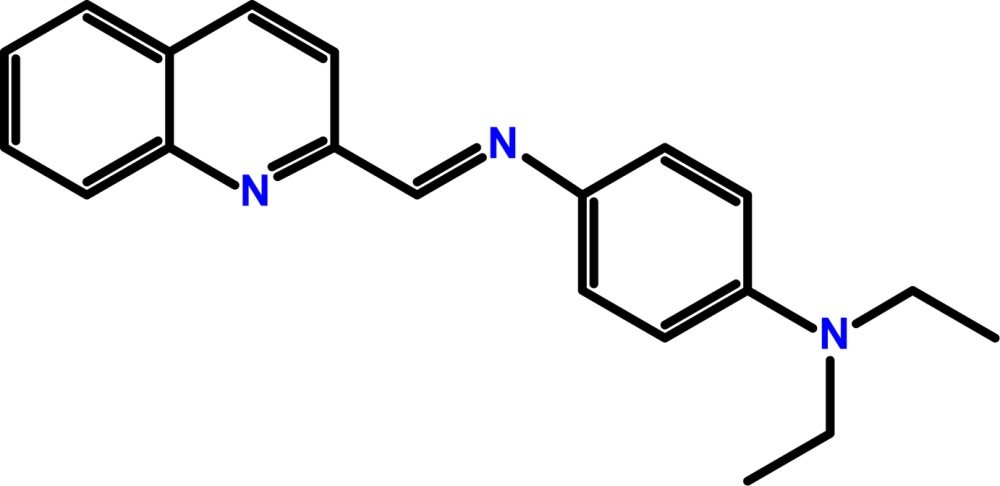


## Experimental   

### Crystal data  

C_20_H_21_N_3_
*M* *_r_* = 303.40
Orthorhombic, 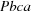

*a* = 20.354 (5) Å
*b* = 7.534 (5) Å
*c* = 21.801 (5) Å
*V* = 3343 (2) Å^3^
*Z* = 8
Mo *K*α radiation
μ = 0.07 mm^−1^
*T* = 100 K
0.27 × 0.21 × 0.16 mm

### Data collection  

Bruker SMART APEX CCD diffractometer
Absorption correction: multi-scan (*SADABS*; Sheldrick, 2008*a* ▸) *T*_min_ = 0.981, *T*_max_ = 0.989
14928 measured reflections
2937 independent reflections
1912 reflections with *I* > 2σ(*I*)
*R*_int_ = 0.146

### Refinement  

*R*[*F* ^2^ > 2σ(*F* ^2^)] = 0.084
*wR*(*F* ^2^) = 0.195
*S* = 1.09
2937 reflections
212 parameters
H atoms treated by a mixture of independent and constrained refinement
Δρ_max_ = 0.32 e Å^−3^
Δρ_min_ = −0.23 e Å^−3^

### 

Data collection: *SMART* (Bruker, 2003 ▸); cell refinement: *SAINT* (Bruker, 2003 ▸); data reduction: *SAINT*; program(s) used to solve structure: *SIR97* (Altomare *et al.*, 1999 ▸); program(s) used to refine structure: *SHELXL97* (Sheldrick, 2008*b* ▸); molecular graphics: *DIAMOND* (Brandenberg & Putz, 2005 ▸); software used to prepare material for publication: *DIAMOND*.

## Supplementary Material

Crystal structure: contains datablock(s) global, I. DOI: 10.1107/S2056989014027108/hg5416sup1.cif

Structure factors: contains datablock(s) I. DOI: 10.1107/S2056989014027108/hg5416Isup2.hkl

Supporting information file. DOI: 10.1107/S2056989014027108/hg5416Isup3.cml

**Fig. 1.**
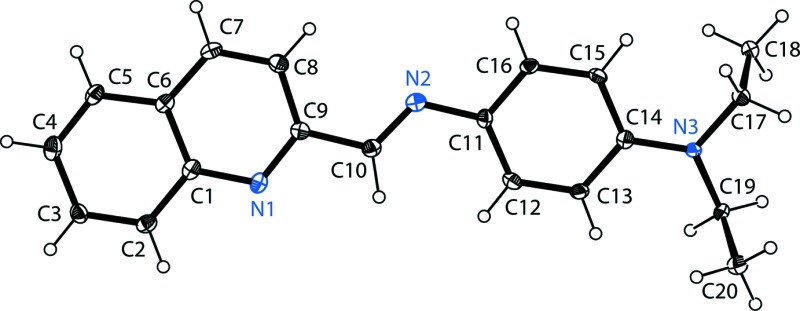
The molecular conformation and atom-numbering scheme for the title compound, with non-H atoms drawn as 40% probability displacement ellipsoids.

**Fig. 2.**
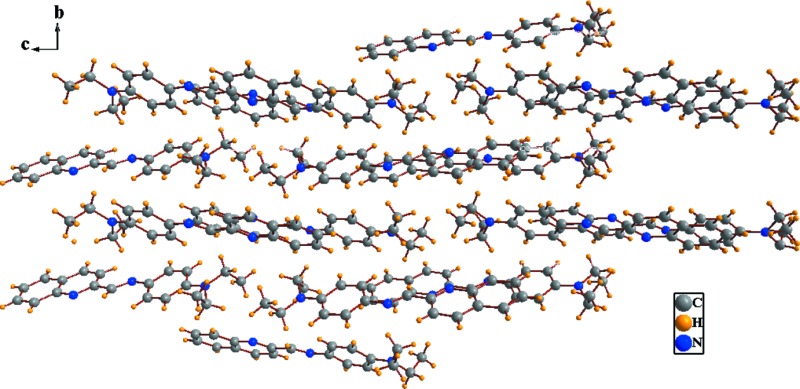
The molecular packing viewed along the a direction.

. DOI: 10.1107/S2056989014027108/hg5416fig1.tif
The mol­ecular conformation and atom-numbering scheme for the title compound, with non-H atoms drawn as 40% probability displacement ellipsoids.

a . DOI: 10.1107/S2056989014027108/hg5416fig2.tif
The mol­ecular packing viewed along the *a* direction.

CCDC reference: 1038674

Additional supporting information:  crystallographic information; 3D view; checkCIF report

## Figures and Tables

**Table 1:** Hydrogen-bond geometry (, ) *Cg*1, *Cg*2 and *Cg*3 are the centroids of the N1/C1/C6C9, C1C16 and C11C16 rings, respectively.

*D*H*A*	*D*H	H*A*	*D* *A*	*D*H*A*
C5H5*Cg*2^i^	0.93	2.99	3.705(5)	135
C7H7*Cg*1^i^	0.93	2.90	3.612(5)	135
C13H13*Cg*3^ii^	0.93	2.84	3.588(5)	138
C15H15*Cg*2^iii^	0.93	2.89	3.686(5)	145
C18H18*A* *Cg*1^iii^	0.96	2.95	3.625(5)	128

Symmetry codes: (i) 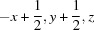; (ii) 
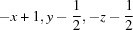
; (iii) 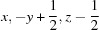.

## supplementary crystallographic information

### S1. Comment

Quinoline derivatives of Schiff bases are important building blocks of many important compounds widely used in biological applications such as antioxidative and anticancer and fluorescent probe agents in industry and in coordination chemistry (Motswainyana *et al.*, 2013; Das *et al.*, 2013; Song *et al.*, 2011; Jursic *et al.*, 2002). The present work is part of an ongoing structural study of Schiff base metal complexes (Faizi & Hussain, 2014; Faizi & Sen, 2014; Faizi *et al.* 2014) and we report here the structure of *N*^1^,*N*^1^-diethyl-*N*^4^-[(quinolin-2-yl)methylidene]benzene-1,4-diamine (DQMBD). There are very few examples similar to title compound and their metal complex have been reported in the literature (Patra & Goldberg 2003; Gonzalez *et al.*, 2012). The synthesis of DQMBD by condensation of 2-quinolinecarboxaldehyde and *N*^1^,*N*^1^-diethyl-*p*-phenylenediamine has not previously been reported. In the title compound (Fig. 1) DQMBD has non planar structure, the dihedral angle between the quinolinyl and *p*phenylenediamine rings is 9.40 (4)°. In the crystal, molecules are connected by C—H···π, generating a chain extending along the *a* axis direction. In the crystal molecules are connected by C—H···π, giving an overall two-dimensional layered structure lying parallel to (100) is given in Fig. 2.

### S2. Experimental

100 mg (1 mmol) of *N*^1^,*N*^1^-diethyl-*p*-phenylenediamine were dissolved in 10 ml of absolute ethanol. To this solution, 96 mg (1 mmol) of 2-quinolinecarboxaldehyde in 5 ml of absolute ethanol was dropwisely added under stirring. Then, this mixture was stirred for 10 min, two drops of glacial acetic acid were then added and the mixture was further refluxed for 2h. The resulting yellow precipitate was recovered by filtration, washed several times with a small portions of EtOH and then with diethyl ether to give 160 mg (88%) of *N*^1^,*N*^1^-diethyl-*N*^4^-(quinolin-2-ylmethylene)benzene-1,4-diamine (DQMBD). The crystal of the title compound suitable for X-ray analysis was obtained within 4 days by slow evaporation of the EtOH solvent.

### S3. Refinement

the N-bound H-atoms were located in difference Fourier maps,and their positions were then held fixed. All H-atoms were positioned geometrically and refined using a riding model with C—H = 0.92–0.93 Å and *U*_iso_(H) = 1.2*U*_eq_(C).

### Crystal data

**Table:**

C_20_H_21_N_3_	*F*(000) = 1296
*M**_r_* = 303.40	*D*_x_ = 1.206 Mg m^−^^3^
Orthorhombic, *P**b**c**a*	Mo *K*α radiation, λ = 0.71073 Å
Hall symbol: -P 2ac 2ab	Cell parameters from 1765 reflections
*a* = 20.354 (5) Å	θ = 2.7–27.5°
*b* = 7.534 (5) Å	µ = 0.07 mm^−^^1^
*c* = 21.801 (5) Å	*T* = 100 K
*V* = 3343 (2) Å^3^	Needle, yellow
*Z* = 8	0.27 × 0.21 × 0.16 mm

### Data collection

**Table:**

Bruker SMART APEX CCD diffractometer	2937 independent reflections
Radiation source: fine-focus sealed tube	1912 reflections with *I* > 2σ(*I*)
Graphite monochromator	*R*_int_ = 0.146
/w–scans	θ_max_ = 25.0°, θ_min_ = 1.9°
Absorption correction: multi-scan (*SADABS*; Sheldrick, 2008a)	*h* = −24→23
*T*_min_ = 0.981, *T*_max_ = 0.989	*k* = −8→8
14928 measured reflections	*l* = −18→25

### Refinement

**Table:**

Refinement on *F*^2^	Primary atom site location: structure-invariant direct methods
Least-squares matrix: full	Secondary atom site location: difference Fourier map
*R*[*F*^2^ > 2σ(*F*^2^)] = 0.084	Hydrogen site location: inferred from neighbouring sites
*wR*(*F*^2^) = 0.195	H atoms treated by a mixture of independent and constrained refinement
*S* = 1.09	*w* = 1/[σ^2^(*F*_o_^2^) + (0.0662*P*)^2^ + 3.082*P*] where *P* = (*F*_o_^2^ + 2*F*_c_^2^)/3
2937 reflections	(Δ/σ)_max_ < 0.001
212 parameters	Δρ_max_ = 0.32 e Å^−^^3^
0 restraints	Δρ_min_ = −0.23 e Å^−^^3^

### Special details

**Table:**

Geometry. All e.s.d.'s (except the e.s.d. in the dihedral angle between two l.s. planes) are estimated using the full covariance matrix. The cell e.s.d.'s are taken into account individually in the estimation of e.s.d.'s in distances, angles and torsion angles; correlations between e.s.d.'s in cell parameters are only used when they are defined by crystal symmetry. An approximate (isotropic) treatment of cell e.s.d.'s is used for estimating e.s.d.'s involving l.s. planes.
Refinement. Refinement of *F*^2^ against ALL reflections. The weighted *R*-factor *wR* and goodness of fit *S* are based on *F*^2^, conventional *R*-factors *R* are based on *F*, with *F* set to zero for negative *F*^2^. The threshold expression of *F*^2^ > σ(*F*^2^) is used only for calculating *R*-factors(gt) *etc*. and is not relevant to the choice of reflections for refinement. *R*-factors based on *F*^2^ are statistically about twice as large as those based on *F*, and *R*- factors based on ALL data will be even larger.

### Fractional atomic coordinates and isotropic or equivalent isotropic displacement parameters (Å^2^)

**Table:**

	*x*	*y*	*z*	*U*_iso_*/*U*_eq_	
C1	0.35623 (17)	−0.0493 (5)	0.07353 (16)	0.0221 (9)	
C2	0.38904 (17)	−0.1200 (5)	0.12533 (16)	0.0243 (9)	
H2	0.4301	−0.1726	0.1205	0.029*	
C3	0.36114 (17)	−0.1119 (5)	0.18233 (16)	0.0241 (9)	
H3	0.3830	−0.1600	0.2159	0.029*	
C4	0.29920 (19)	−0.0306 (5)	0.19034 (17)	0.0292 (10)	
H4	0.2806	−0.0249	0.2292	0.035*	
C5	0.26640 (18)	0.0397 (5)	0.14123 (16)	0.0246 (9)	
H5	0.2257	0.0930	0.1472	0.029*	
C6	0.29346 (17)	0.0326 (4)	0.08169 (16)	0.0195 (8)	
C7	0.26231 (17)	0.0998 (5)	0.02881 (16)	0.0233 (9)	
H7	0.2216	0.1552	0.0322	0.028*	
C8	0.29143 (17)	0.0843 (5)	−0.02734 (16)	0.0237 (9)	
H8	0.2705	0.1256	−0.0625	0.028*	
C9	0.35419 (17)	0.0040 (5)	−0.03101 (16)	0.0204 (8)	
C10	0.38967 (19)	−0.0092 (5)	−0.08980 (17)	0.0232 (9)	
C11	0.39650 (17)	0.0327 (5)	−0.19610 (15)	0.0208 (8)	
C12	0.45481 (18)	−0.0590 (5)	−0.20748 (16)	0.0229 (8)	
H12	0.4741	−0.1235	−0.1759	0.027*	
C13	0.48440 (17)	−0.0565 (5)	−0.26392 (15)	0.0225 (9)	
H13	0.5233	−0.1190	−0.2696	0.027*	
C14	0.45734 (17)	0.0384 (5)	−0.31347 (15)	0.0197 (8)	
C15	0.39852 (16)	0.1316 (5)	−0.30210 (16)	0.0208 (8)	
H15	0.3792	0.1971	−0.3335	0.025*	
C16	0.36925 (17)	0.1269 (5)	−0.24513 (15)	0.0199 (8)	
H16	0.3301	0.1883	−0.2391	0.024*	
C17	0.46058 (17)	0.1451 (5)	−0.42064 (15)	0.0226 (9)	
H17A	0.4951	0.1662	−0.4505	0.027*	
H17B	0.4464	0.2595	−0.4051	0.027*	
C18	0.40284 (17)	0.0555 (5)	−0.45265 (16)	0.0255 (9)	
H18A	0.3874	0.1299	−0.4854	0.038*	
H18B	0.3680	0.0367	−0.4237	0.038*	
H18C	0.4167	−0.0567	−0.4691	0.038*	
C19	0.54187 (16)	−0.0777 (5)	−0.38531 (15)	0.0213 (8)	
H19A	0.5381	−0.1130	−0.4280	0.026*	
H19B	0.5382	−0.1839	−0.3604	0.026*	
C20	0.60919 (18)	0.0044 (6)	−0.37512 (18)	0.0308 (10)	
H20A	0.6426	−0.0802	−0.3856	0.046*	
H20B	0.6137	0.0375	−0.3328	0.046*	
H20C	0.6137	0.1078	−0.4005	0.046*	
N1	0.38663 (14)	−0.0612 (4)	0.01724 (13)	0.0227 (8)	
N2	0.36253 (14)	0.0420 (4)	−0.13964 (13)	0.0227 (7)	
N3	0.48728 (14)	0.0418 (4)	−0.37025 (12)	0.0201 (7)	
H10	0.4353 (17)	−0.053 (4)	−0.0845 (14)	0.015 (9)*	

### Atomic displacement parameters (Å^2^)

**Table:**

	*U*^11^	*U*^22^	*U*^33^	*U*^12^	*U*^13^	*U*^23^
C1	0.021 (2)	0.020 (2)	0.025 (2)	−0.0063 (16)	0.0031 (16)	−0.0065 (16)
C2	0.019 (2)	0.024 (2)	0.030 (2)	−0.0016 (16)	0.0000 (16)	−0.0058 (17)
C3	0.025 (2)	0.028 (2)	0.0193 (19)	−0.0033 (17)	−0.0025 (16)	−0.0011 (16)
C4	0.030 (2)	0.033 (2)	0.025 (2)	−0.0110 (18)	0.0055 (17)	−0.0053 (18)
C5	0.020 (2)	0.027 (2)	0.027 (2)	−0.0042 (16)	0.0055 (16)	−0.0068 (17)
C6	0.0166 (19)	0.0157 (19)	0.0261 (19)	−0.0065 (15)	0.0018 (15)	−0.0037 (15)
C7	0.016 (2)	0.021 (2)	0.033 (2)	0.0013 (15)	0.0014 (16)	−0.0035 (17)
C8	0.023 (2)	0.023 (2)	0.025 (2)	−0.0025 (16)	−0.0044 (16)	−0.0014 (16)
C9	0.022 (2)	0.015 (2)	0.0239 (19)	−0.0012 (15)	−0.0022 (15)	−0.0019 (15)
C10	0.022 (2)	0.022 (2)	0.026 (2)	0.0014 (16)	0.0010 (16)	0.0003 (16)
C11	0.023 (2)	0.023 (2)	0.0170 (18)	−0.0011 (16)	0.0016 (15)	−0.0021 (15)
C12	0.026 (2)	0.021 (2)	0.0212 (19)	0.0028 (16)	−0.0063 (16)	0.0007 (16)
C13	0.019 (2)	0.025 (2)	0.023 (2)	0.0060 (16)	−0.0012 (15)	−0.0025 (16)
C14	0.020 (2)	0.020 (2)	0.0191 (18)	−0.0048 (15)	−0.0011 (15)	−0.0034 (15)
C15	0.0205 (19)	0.022 (2)	0.0196 (19)	0.0039 (16)	−0.0061 (15)	0.0010 (16)
C16	0.0134 (18)	0.024 (2)	0.0224 (19)	−0.0006 (15)	−0.0029 (15)	−0.0050 (16)
C17	0.022 (2)	0.025 (2)	0.0207 (19)	−0.0001 (16)	−0.0018 (15)	0.0002 (16)
C18	0.025 (2)	0.031 (2)	0.0203 (19)	0.0025 (17)	0.0002 (16)	0.0029 (16)
C19	0.019 (2)	0.027 (2)	0.0175 (19)	0.0035 (15)	0.0036 (15)	−0.0015 (16)
C20	0.026 (2)	0.034 (2)	0.032 (2)	0.0057 (18)	0.0025 (17)	0.0039 (19)
N1	0.0247 (18)	0.0214 (19)	0.0221 (17)	−0.0012 (13)	0.0046 (13)	−0.0022 (13)
N2	0.0211 (17)	0.0194 (17)	0.0277 (18)	−0.0012 (13)	−0.0012 (13)	−0.0017 (14)
N3	0.0177 (17)	0.0266 (18)	0.0161 (15)	0.0078 (13)	−0.0002 (12)	0.0001 (13)

### Geometric parameters (Å, º)

**Table:**

C1—N1	1.377 (4)	C12—C13	1.370 (5)
C1—C2	1.416 (5)	C12—H12	0.9300
C1—C6	1.430 (5)	C13—C14	1.408 (5)
C2—C3	1.368 (5)	C13—H13	0.9300
C2—H2	0.9300	C14—N3	1.380 (4)
C3—C4	1.412 (5)	C14—C15	1.410 (5)
C3—H3	0.9300	C15—C16	1.378 (5)
C4—C5	1.368 (5)	C15—H15	0.9300
C4—H4	0.9300	C16—H16	0.9300
C5—C6	1.411 (5)	C17—N3	1.452 (4)
C5—H5	0.9300	C17—C18	1.525 (5)
C6—C7	1.410 (5)	C17—H17A	0.9700
C7—C8	1.365 (5)	C17—H17B	0.9700
C7—H7	0.9300	C18—H18A	0.9600
C8—C9	1.416 (5)	C18—H18B	0.9600
C8—H8	0.9300	C18—H18C	0.9600
C9—N1	1.336 (4)	C19—N3	1.467 (4)
C9—C10	1.474 (5)	C19—C20	1.519 (5)
C10—N2	1.278 (5)	C19—H19A	0.9700
C10—H10	0.99 (3)	C19—H19B	0.9700
C11—C12	1.396 (5)	C20—H20A	0.9600
C11—C16	1.398 (5)	C20—H20B	0.9600
C11—N2	1.413 (4)	C20—H20C	0.9600
			
N1—C1—C2	118.3 (3)	N3—C14—C13	121.7 (3)
N1—C1—C6	122.7 (3)	N3—C14—C15	121.6 (3)
C2—C1—C6	119.0 (3)	C13—C14—C15	116.8 (3)
C3—C2—C1	120.8 (3)	C16—C15—C14	120.8 (3)
C3—C2—H2	119.6	C16—C15—H15	119.6
C1—C2—H2	119.6	C14—C15—H15	119.6
C2—C3—C4	120.2 (3)	C15—C16—C11	122.1 (3)
C2—C3—H3	119.9	C15—C16—H16	119.0
C4—C3—H3	119.9	C11—C16—H16	119.0
C5—C4—C3	120.4 (3)	N3—C17—C18	113.4 (3)
C5—C4—H4	119.8	N3—C17—H17A	108.9
C3—C4—H4	119.8	C18—C17—H17A	108.9
C4—C5—C6	121.0 (4)	N3—C17—H17B	108.9
C4—C5—H5	119.5	C18—C17—H17B	108.9
C6—C5—H5	119.5	H17A—C17—H17B	107.7
C7—C6—C5	124.3 (3)	C17—C18—H18A	109.5
C7—C6—C1	117.1 (3)	C17—C18—H18B	109.5
C5—C6—C1	118.7 (3)	H18A—C18—H18B	109.5
C8—C7—C6	120.5 (3)	C17—C18—H18C	109.5
C8—C7—H7	119.8	H18A—C18—H18C	109.5
C6—C7—H7	119.8	H18B—C18—H18C	109.5
C7—C8—C9	118.6 (3)	N3—C19—C20	113.6 (3)
C7—C8—H8	120.7	N3—C19—H19A	108.8
C9—C8—H8	120.7	C20—C19—H19A	108.8
N1—C9—C8	124.0 (3)	N3—C19—H19B	108.8
N1—C9—C10	114.7 (3)	C20—C19—H19B	108.8
C8—C9—C10	121.3 (3)	H19A—C19—H19B	107.7
N2—C10—C9	120.5 (3)	C19—C20—H20A	109.5
N2—C10—H10	127.2 (18)	C19—C20—H20B	109.5
C9—C10—H10	112.2 (18)	H20A—C20—H20B	109.5
C12—C11—C16	116.9 (3)	C19—C20—H20C	109.5
C12—C11—N2	126.5 (3)	H20A—C20—H20C	109.5
C16—C11—N2	116.5 (3)	H20B—C20—H20C	109.5
C13—C12—C11	121.8 (3)	C9—N1—C1	117.1 (3)
C13—C12—H12	119.1	C10—N2—C11	120.9 (3)
C11—C12—H12	119.1	C14—N3—C17	121.6 (3)
C12—C13—C14	121.6 (3)	C14—N3—C19	121.6 (3)
C12—C13—H13	119.2	C17—N3—C19	116.3 (3)
C14—C13—H13	119.2		
			
N1—C1—C2—C3	−180.0 (3)	C12—C13—C14—C15	0.4 (5)
C6—C1—C2—C3	0.5 (5)	N3—C14—C15—C16	−180.0 (3)
C1—C2—C3—C4	−0.7 (6)	C13—C14—C15—C16	−0.7 (5)
C2—C3—C4—C5	0.4 (6)	C14—C15—C16—C11	0.9 (5)
C3—C4—C5—C6	0.1 (6)	C12—C11—C16—C15	−0.7 (5)
C4—C5—C6—C7	179.1 (3)	N2—C11—C16—C15	178.5 (3)
C4—C5—C6—C1	−0.2 (5)	C8—C9—N1—C1	0.4 (5)
N1—C1—C6—C7	1.1 (5)	C10—C9—N1—C1	178.8 (3)
C2—C1—C6—C7	−179.5 (3)	C2—C1—N1—C9	179.0 (3)
N1—C1—C6—C5	−179.5 (3)	C6—C1—N1—C9	−1.6 (5)
C2—C1—C6—C5	−0.1 (5)	C9—C10—N2—C11	178.9 (3)
C5—C6—C7—C8	−178.7 (3)	C12—C11—N2—C10	13.1 (6)
C1—C6—C7—C8	0.6 (5)	C16—C11—N2—C10	−166.1 (3)
C6—C7—C8—C9	−1.8 (5)	C13—C14—N3—C17	−177.7 (3)
C7—C8—C9—N1	1.3 (5)	C15—C14—N3—C17	1.5 (5)
C7—C8—C9—C10	−177.1 (3)	C13—C14—N3—C19	11.2 (5)
N1—C9—C10—N2	177.1 (3)	C15—C14—N3—C19	−169.6 (3)
C8—C9—C10—N2	−4.5 (5)	C18—C17—N3—C14	−79.2 (4)
C16—C11—C12—C13	0.4 (5)	C18—C17—N3—C19	92.3 (4)
N2—C11—C12—C13	−178.8 (3)	C20—C19—N3—C14	−94.8 (4)
C11—C12—C13—C14	−0.2 (6)	C20—C19—N3—C17	93.7 (4)
C12—C13—C14—N3	179.6 (3)		

### Hydrogen-bond geometry (Å, º)

Cg1, Cg2 and Cg3 are the centroids of the N1/C1/C6–C9, C1–C16 and C11–C16 rings, respectively.

**Table:**

*D*—H···*A*	*D*—H	H···*A*	*D*···*A*	*D*—H···*A*
C5—H5···*Cg*2^i^	0.93	2.99	3.705 (5)	135
C7—H7···*Cg*1^i^	0.93	2.90	3.612 (5)	135
C13—H13···*Cg*3^ii^	0.93	2.84	3.588 (5)	138
C15—H15···*Cg*2^iii^	0.93	2.89	3.686 (5)	145
C18—H18*A*···*Cg*1^iii^	0.96	2.95	3.625 (5)	128

Symmetry codes: (i) −*x*+1/2, *y*+1/2, *z*; (ii) −*x*+1, *y*−1/2, −*z*−1/2; (iii) *x*, −*y*+1/2, *z*−1/2.
