# Supplementary figures and images for: Crystal structure of N 1,N 1-diethyl-N 4-[(quinolin-2-yl)methyl­idene]benzene-1,4-di­amine
Source: Acta Crystallogr E Crystallogr Commun. 2015 Jan 1;71(Pt 1):o49–50. doi: 10.1107/S2056989014027108 (PMC4331901; doi:10.1107/S2056989014027108)

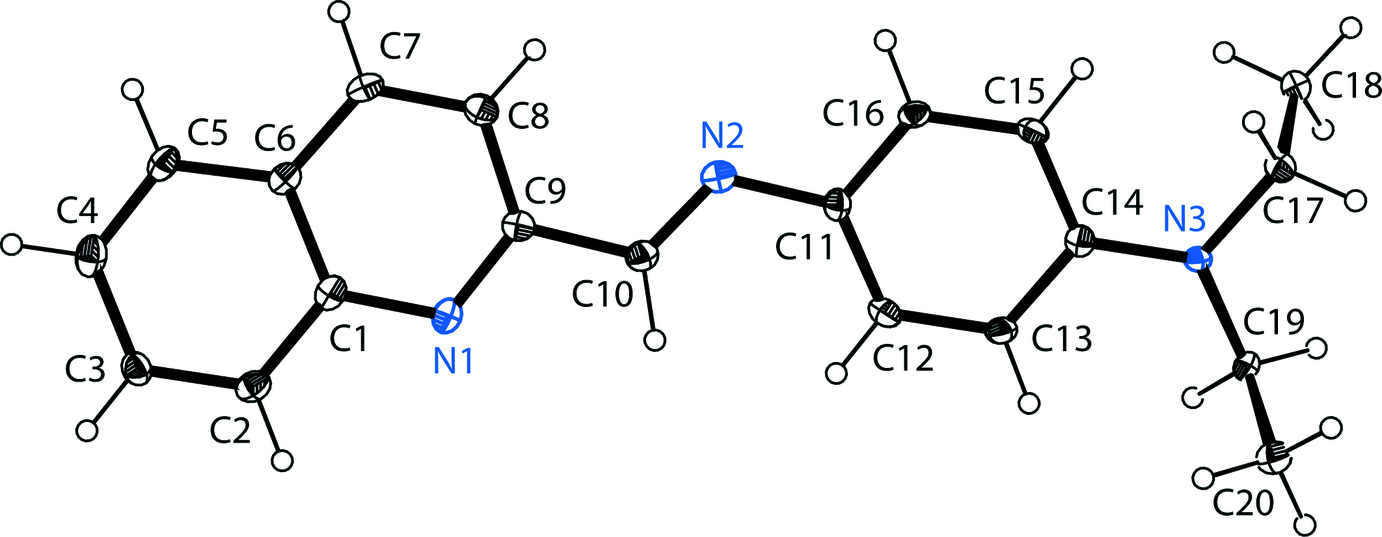

Supplement: Supplementary file 4 [file e-71-00o49-fig1.tif]

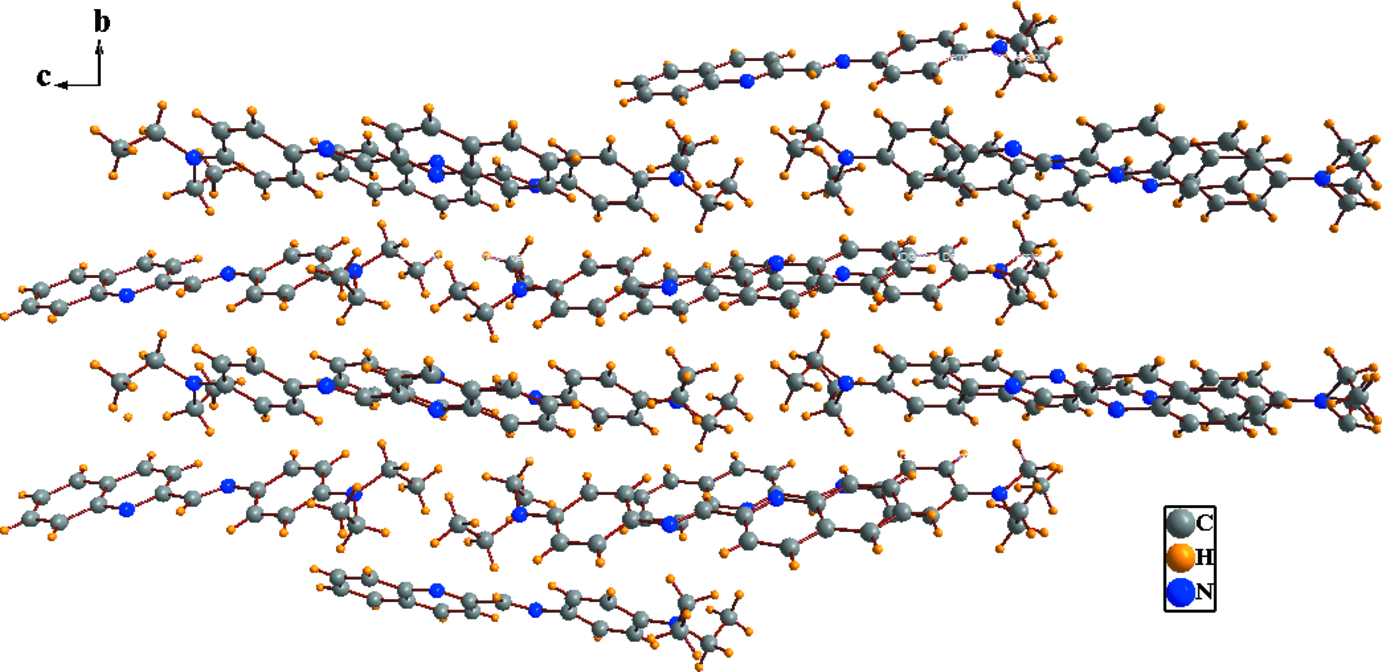

Supplement: Supplementary file 5 [file e-71-00o49-fig2.tif]
